# Inhibiting mtDNA transcript translation alters Alzheimer's disease‐associated biology

**DOI:** 10.1002/alz.14275

**Published:** 2024-10-23

**Authors:** Alexander P. Gabrielli, Lesya Novikova, Amol Ranjan, Xiaowan Wang, Nicholas J. Ernst, Dhanushki Abeykoon, Anysja Roberts, Annie Kopp, Clayton Mansel, Linlan Qiao, Colton R. Lysaker, Ian W. Wiedling, Heather M. Wilkins, Russell H. Swerdlow

**Affiliations:** ^1^ University of Kansas Alzheimer's Disease Research Center Kansas City Kansas USA; ^2^ Cell Biology and Physiology University of Kansas Medical Center Kansas City Kansas USA; ^3^ Biochemistry and Molecular Biology, University of Kansas Medical Center Kansas City Kansas USA; ^4^ Neurology the University of Kansas Medical Center Kansas City Kansas USA

**Keywords:** Alzheimer's disease, amyloid, mitochondria, neurons, translation

## Abstract

**INTRODUCTION:**

Alzheimer's disease (AD) features changes in mitochondrial structure and function. Investigators debate where to position mitochondrial pathology within the chronology and context of other AD features.

**METHODS:**

To address whether mitochondrial dysfunction alters AD‐implicated genes and proteins, we treated SH‐SY5Y cells and induced pluripotent stem cell (iPSC)‐derived neurons with chloramphenicol, an antibiotic that inhibits mtDNA‐generated transcript translation. We characterized adaptive, AD‐associated gene, and AD‐associated protein responses.

**RESULTS:**

SH‐SY5Y cells and iPSC neurons responded to mtDNA transcript translation inhibition by increasing mtDNA copy number and transcription. Nuclear‐expressed respiratory chain mRNA and protein levels also changed. There were AD‐consistent concordant and model‐specific changes in amyloid precursor protein, beta amyloid, apolipoprotein E, tau, and α‐synuclein biology.

**DISCUSSION:**

Primary mitochondrial dysfunction induces compensatory organelle responses, changes nuclear gene expression, and alters the biology of AD‐associated genes and proteins in ways that may recapitulate brain aging and AD molecular phenomena.

**Highlights:**

- In AD, mitochondrial dysfunction could represent a disease cause or consequence.
- We inhibited mitochondrial translation in human neuronal cells and neurons.
- Mitochondrial and nuclear gene expression shifted in adaptive‐consistent patterns.
- APP, Aβ, APOE, tau, and α‐synuclein biology changed in AD‐consistent patterns.
- Mitochondrial stress creates an environment that promotes AD pathology.

## 1 BACKGROUND

Persons with Alzheimer's disease (AD) have altered mitochondrial structure and function.^1^ Investigators debate where to position these perturbations within proposed AD molecular cascades.^2^, ^3^ The amyloid cascade hypothesis predicts changes in mitochondrial biology are directly mediated by fibrillar, protofibrillar, oligomeric, or monomeric beta amyloid (Aβ) protein, or indirectly through an effect of some other Aβ‐altered biology.^4^, ^5^, ^6^ Alternatively, a mitochondrial cascade hypothesis predicts perturbed mitochondria may play an upstream role, and in fact trigger the classic histopathology changes we associate with the disease.^7^, ^8^

While the brains of transgenic models that express mutant amyloid precursor protein (APP) and presenilin genes develop damaged and dysfunctional mitochondria,^9^, ^10^, ^11^ these models are not suitable for testing the mitochondrial cascade hypothesis. Testing mitochondrial cascade hypothesis predictions requires models of primary mitochondrial dysfunction, but there are many ways to induce mitochondrial dysfunction and different approaches can produce different responses. We previously reported data from models of primary mitochondrial dysfunction that arose through mitochondrial DNA (mtDNA) transfer from AD patients to neuronal cell lines depleted of their endogenous mtDNA (ρ0 cells),^1^, ^12^, ^13^ and more recently simply from ρ0 cells themselves; these models exhibit changes in AD‐associated biologies including changes in Aβ, tau, and apolipoprotein E (*APOE* [gene]; apoE [protein]) levels.^14^, ^15^, ^16^

Here, we evaluate the impact of primary mitochondrial dysfunction that results from the inhibition of mtDNA‐generated transcript translation. The premise for this approach is informed by a report that genetic variation in the *PTCD1* gene, which encodes a protein that participates in mitochondrial ribosome translation, affects AD risk.^17^ From perhaps a more generalizable perspective, a recent bioinformatics analysis of AD brain transcriptomes and proteomes also reported profound perturbation of a mitochondrial translation domain.^18^ Our model leveraged chloramphenicol, an antibiotic that inhibits mitochondrial but not cytoplasmic ribosome translation.^19^ We tested the impact of chloramphenicol‐induced mitochondrial translation inhibition in both a human neuronal cell line and in human forebrain neurons generated from induced pluripotent stem cells (iPSCs). We specifically considered how chloramphenicol‐mediated inhibition of mtDNA transcript translation affected the biology of AD‐associated, nuclear‐expressed genes and proteins.

**RESEARCH IN CONTEXT**

1. **Systematic review**: Mitochondrial dysfunction could represent an AD cause or consequence, but traditional AD models only test the latter possibility. To test whether primary mitochondrial dysfunction could initiate AD‐consistent molecular changes, the authors created novel models of primary mitochondrial dysfunction by inhibiting neuronal cell and induced pluripotent stem cell (iPSC)‐derived neuron mitochondrial gene transcript translation. Both responded by increasing mtDNA copy number and transcription, and nuclear‐expressed respiratory chain mRNA and protein levels changed. There were AD‐consistent changes in APP, Aβ, APOE, tau, and α‐synuclein biology.
2. **Interpretation**: Mitochondrial stress induces compensatory responses, changes nuclear gene expression, and alters AD‐associated biology in ways that prime AD molecular phenomena. Our findings are consistent with predictions made by a mitochondrial cascade hypothesis that proposes primary mitochondrial dysfunction initiates AD.
3. **Future directions**: These mitochondrial translation inhibition models will facilitate studies that advance our understanding of mitochondria‐nuclear communication and inform the integrity of the mitochondrial cascade hypothesis.

## 2 METHODS

### 2.1 Human induced‐pluripotent stem cells

A fibroblast‐derived, human *APOE ε*3/*ε*3 induced pluripotent stem cell (iPSC) line was obtained from Jackson labs (JIPSC1268). The iPSCs were cultured on Matrigel‐coated culture ware using a feeder free system. Passaging was performed using ReLeSR (STEMCELL Technologies) to select for non‐differentiated colonies.

### 2.2 iPSC‐derived forebrain neurons

Culture ware was precoated with Corning Matrigel hESC‐Qualified Matrix (Fisher Scientific 08‐774‐552) dissolved in DMEM/F12 with HEPES (Thermo Fisher 10565018). The iPSCs were induced into neural progenitor cells (NPCs) using the embryoid body protocol method outlined by STEMCELL Technologies. NPCs were grown in STEMdiff Neural Progenitor Medium (STEMCELL Technologies 05833) supplemented with 0.5% penicillin‐streptomycin by volume (Thermo Fisher 15140122). The NPC media was changed daily. NPCs were grown and maintained at 12 passages or fewer. Upon reaching ≈80% confluency, NPCs were differentiated into forebrain neurons with the STEMdiff Forebrain Neuron Differentiation Kit (STEMCELL Technologies 08600). The forebrain neuron differentiation medium was replaced every other day. After 1 week, forebrain neurons were replated and matured in BrainPhys Neuronal Medium (STEMCELL Technologies 05790) supplemented with NeuroCult SM1 Neuronal Supplement (STEMCELL Technologies 05711), N2 Supplement‐A (STEMCELL Technologies 07152), ascorbic acid, Human Recombinant BDNF (STEMCELL Technologies 78005.1), Human Recombinant GDNF (STEMCELL Technologies 78058.1), dibutyryl‐cAMP (STEMCELL Technologies 100‐0244), and penicillin‐streptomycin. Neurons matured for 2 to 3 weeks with half‐media changes every other day. Cultures were maintained in a humidified incubator at 37°C and 5% CO_2_. NPCs and neurons were replated with Accutase (STEMCELL Technologies 07920).

### 2.3 SH‐SY5Y cells with or without mtDNA

SH‐SY5Y cells with (ρ+) or without (ρ0) mtDNA were maintained in high‐glucose, L‐glutamine Dulbecco's Modified Eagle Medium (DMEM) supplemented with 10% fetal bovine serum (FBS), 100 µg/mL sodium pyruvate, 50 µg/mL uridine, and 1% penicillin‐streptomycin. The ρ0 cells were generated through chronic ethidium bromide exposure.^20^ Upon achieving complete mtDNA depletion, ethidium bromide was no longer added to the medium and reversion to ρ+ status did not occur.

Our SH‐SY5Y ρ+ and ρ0 cell lines were seeded at 25% to 50% confluency and maintained side‐by‐side in a humidified incubator at 37°C and 5% CO_2_. Cells were harvested at ≈80% confluency for subsequent analyses.

### 2.4 Chloramphenicol treatment

SH‐SY5Y cells in their undifferentiated state were placed in 200 µg/mL chloramphenicol (Sigma Aldrich C3175‐100MG) for 1 week, and this concentration was maintained with each medium change. After maturation, iPSC‐derived neurons were maintained in BrainPhys Neuronal Medium and supplements plus 200 µg/mL chloramphenicol. Control and chloramphenicol‐treated neurons were subjected to half‐media changes every other day for 1 week, and fresh chloramphenicol was added to reach 200 µg/mL with each medium change. The 200 µg/mL dosage was guided by the literature, in conjunction with our own dose‐response assays.^21^

### 2.5 Immunoblotting

To generate whole cell protein lysates, SH‐SY5Y cells and neurons were washed with ice‐cold phosphate‐buffered saline (PBS) and scraped into RIPA buffer supplemented with Halt Protease and Phosphatase Inhibitor Cocktail (Thermo Fisher 1861284). The resulting suspensions were vigorously vortexed, incubated on ice for 15 minutes, vortexed again, and centrifuged at 14,000 rcf for 15 minutes at 4°C. Supernatants were collected in a fresh receptacle for further work.

For western blot immunochemistry, protein concentrations were measured with a bicinchoninic acid (BCA) assay. Samples were diluted to allow for the addition of 80 to 100 µg of protein per well and boiled with 1X Laemmli‐SDS buffer. Samples were run alongside a pre‐stained protein ladder (10 to 180 kDa, Thermo Fisher, 26616). Following gel electrophoresis, we performed wet transfers to polyvinylidene difluoride (PVDF) membranes, which were blocked for 1 hour with bovine serum albumin (BSA) and maintained overnight in primary antibody solution at 4°C. After imaging, membranes were stripped with Restore PLUS Western Blot Stripping Buffer (Thermo Fisher 46430) and redeveloped. Quantification of targets was performed through densitometry. All targets were normalized to β‐tubulin. Table S1 provides a list of the western blot antibodies.

### 2.6 Fluorescence immunocytochemistry and confocal imaging

For immunofluorescence immunocytochemistry (ICC), iPSC‐derived neurons were cultured in 24‐well plates in a 37°C incubator at 5% CO_2_. Cell culture‐treated poly‐D‐lysine/Laminin Cellware 12 mm round coverslips (Corning, #354087) were placed one per well. Coverslips were pre‐coated in Matrigel for optimal cell attachment and growth. Neurons were plated to 70% to 80% confluency. Neurons were matured for ≈2 weeks, then treated for 1 week with chloramphenicol. Cells were washed in PBS twice for 3 minutes with 500 µL PBS per well. All steps for the staining procedure were performed in a covered 24‐well plate at room temperature. The cells were washed, then fixed in 300 µL of 4% paraformaldehyde in PBS for 20 minutes. The cells were washed again, then permeabilized with 300 µL 0.04% Triton X‐100 in PBS for 30 minutes. After permeabilization, coverslips were lifted and moved to the clean wells.

To prevent non‐specific protein‐protein interactions, cells on coverslips were blocked in PBS containing 10% normal serum/1% BSA/0.3 M glycine/0.03% Triton X‐100 for 1 h. The cells were rinsed with 300 µL of PBS and incubated with primary antibodies mixed in a solution containing 1% normal donkey serum/1% BSA/0.03% Triton X‐100 in PBS (150 µL per well). Incubations were done overnight at 4°C in a humidified chamber.

The plate was protected from light while the cells were incubated with fluorescence‐conjugated secondary antibodies (200 µL per well) for 1.5 hours. The same solution was used for the primary and secondary antibody mix. Cells were washed three times (10 minutes each) in PBS. Nuclei were stained with 4′,6‐diamidino‐2‐phenylindole (DAPI) for 5 minutes before the last wash.

After washing in the wells, coverslips were removed, dried out, and mounted cell‐side down with Prolong Gold antifade reagent (Invitrogen, #P36930) on Superfrost Plus Microscope slides (Fisher Scientific, #12‐550‐15). All slides were viewed and quantified with identical laser settings on a Nikon A1R confocal microscope.

Fluorescence intensity was measured in each cell by sampling the cytosol in two places using ImageJ software. Intensity of cytosolic fluorescence was quantified in at least 30 cells each for both control and chloramphenicol‐treated neurons. Table S2 lists the ICC antibodies.

### 2.7 Enzyme‐linked immunoassay

We used enzyme‐linked immunoassay (ELISA) kits to quantify apoE (EHAPOE; Thermo Fisher) and Aβ42 (KHB3441; Thermo Fisher) proteins in SH‐SY5Y cell and iPSC‐derived neuron conditioned media. The assay plates were prepared per kit instructions and read using a SpectraMax iD5 Multi‐Mode Plate Reader. Standard curves were constructed using a four‐parameter logistic regression.

### 2.8 Cell count and viability assessments

Routine cell counts were performed using a Countess 3 automated cell counter (Thermo Fisher). Viability testing was performed using a trypan blue exclusion assay that relied on manual assessments of harvested cells transferred to a hemocytometer slide.

### 2.9 RNA extraction, cDNA preparation, and real‐time polymerase chain reaction

Neurons were washed with ice‐cold PBS and RNA from cell cultures was isolated using TRIzol reagent, chloroform, and isopropanol extraction. cDNA was prepared from 1 µg input RNA with BIO‐RAD iScript Reverse Transcription Supermix (1708841) for reverse transcription according to their protocol. All quantitative polymerase chain reactions (PCRs) were prepared with Thermo Fisher TaqMan Universal Master Mix II with UNG (4440038) and gene specific Thermo Fisher TaqMan primers (Table S3). Real‐time PCR was performed using a QuantStudio 3 Real‐Time PCR System (96 well) and software. Relative gene expression was determined using the 2^−ΔΔCt^ method. *ACTB* served as the endogenous expresser for all quantitative PCR assays. Each sample was tested in duplicate or greater, and the reported data reflect the average of the replicates.

### 2.10 DNA extraction and determination of mtDNA/nuclear DNA copy number

DNA (mtDNA and nuclear DNA [nucDNA]) was isolated with a QIAamp DNA Mini Kit (QIAGEN 51306). DNA extracts were combined with SYBR Green (Thermo Fisher A25742) and nucDNA or mtDNA‐specific primers. All amplification was performed using the manufacturer's instructions. Primers for amplification were selected as previously described.^22^ A standard curve was generated using nucDNA and mtDNA standards. Estimation of unknowns was determined using the 2^−ΔΔCt^ method in conjunction with a standard curve.

### 2.11 Bioenergetic flux analyses

Oxygen consumption rates (OCRs) and extracellular acidification rates (ECARs) were determined using a Seahorse instrument. ρ+ and ρ0 cell bioenergetic flux analyses were performed as previously described.^14^ Neurons were matured and treated on Seahorse XFe96/XF Pro Cell Culture Microplates (Agilent 103794‐100). BrainPhys Media was replaced with XF DMEM Base Medium (Agilent) at a glucose concentration of 2.5 mM, glutamine concentration of 2 mM, and adjusted to a pH of 7.4. Cells were incubated for 1 hour at 37°C in a humidified incubator before undergoing a predefined mitochondrial respiration protocol (Agilent Seahorse XF Cell Mito Stress Test Kits 103015‐100). Mitochondrial stress testing used the following injection schedule: Baseline, T = 0 minutes; Oligomycin and Hoechst 33342 (Thermo Fisher, 62249), T = 20 minutes; FCCP 0.25 µM, T = 40 minutes; FCCP 0.5 µM, T = 60 minutes; and rotenone plus antimycin A, T = 80 minutes. Plates were normalized by cell count. Cell counts were obtained post‐mitochondrial stress test with a Cytation 1 cell‐imaging multi‐mode microplate reader (BioTek) with Gen5 software. Total cell counts were determined from Hoechst staining and estimated by the software. Wells with fewer than 1000 detected cells were excluded, for a minimum of 20 replicates in each treatment condition.

### 2.12 Maximal velocity assays

SH‐SY5Y cells and iPSC‐derived neurons grown to confluency in T‐75 flasks were treated with chloramphenicol for 1 week. To determine the cytochrome c oxidase (COX) maximal velocity (V_max_) cells were pelleted, washed, and resuspended in 100 µL of PBS. Then, 950 µL of 100 mM PBS (pH 7.4) and 20 µL of 10 mg/mL β‐maltoside (Sigma #D461) were added to a cuvette. The absorbance was read at 550 nm for blanking on a TECAN Infinite 200 PRO microplate reader. Twenty‐five microliters of resuspended, homogenized cells were added to the blank. The cuvette was incubated at 30°C. Reduced cytochrome c (Sigma #C7752) was mixed into the cuvette with a Hamilton syringe (20 µL). The absorbance at 550 nm was read at 6‐second intervals for 2 minutes. Potassium ferricyanide crystals (Sigma #702587) were added to stop the reaction and an additional measurement at 550 nm was made. The latter measurement was subtracted from the previous measurements to remove background. The log of the absorbance was plotted against time to generate the COX V_max_ activity in s^−1^. Each sample was tested twice, and the COX V_max_ was taken as the average of the two. A BCA assay was performed on the cell suspension to obtain total protein.

To quantify the citrate synthase (CS) V_max_ activity, 951 µL of 100 mM Tris pH 8.0 buffer, 4 µL of 10% Triton X‐100 (Sigma #1002841029), and 10 mM DTNB (Sigma #D8130) were mixed in a cuvette. The absorbance of the solution at 412 nm was determined and used for blanking purposes. Fifteen microliters of cell suspension were introduced and the cuvette was incubated for 3 minutes at 30°C. Following incubation, 10 µL of freshly prepared 50 mM oxaloacetate (Sigma #07753, dissolved in 10 mM Tris‐HCl pH 8.0) and 10 µL of 5 mM acetyl‐CoA (Sigma #A2181) were added to the cuvette and mixed. Absorbance was measured at 6‐second intervals at 412 nm for 2 minutes. Absorbance was plotted against time and the resulting slope was multiplied by 60 to obtain the rate/minute and divided by 0.0136, a constant value derived from the Beer‐Lambert Law. The nmol/minute V_max_ was normalized to total protein; the final value represents the average of two measurements.

### 2.13 Statistics

Data analyses were performed with Microsoft Excel and GraphPad Prism statistical software. Group mean comparisons were performed by unpaired, two‐tailed Student's *t*‐test except for those pertaining to V_max_ enzyme activities, which used the non‐parametric Mann‐Whitney *U* test as those data included smaller sample sizes and did not reliably exhibit a normal distribution. For correlation analyses we calculated the Pearson correlation coefficient. Continuous values are portrayed as means ± SEM.

## 3 RESULTS

### 3.1 Chloramphenicol's impact on SH‐SY5Y cell mitochondria and adaptive responses

**FIGURE 1:**
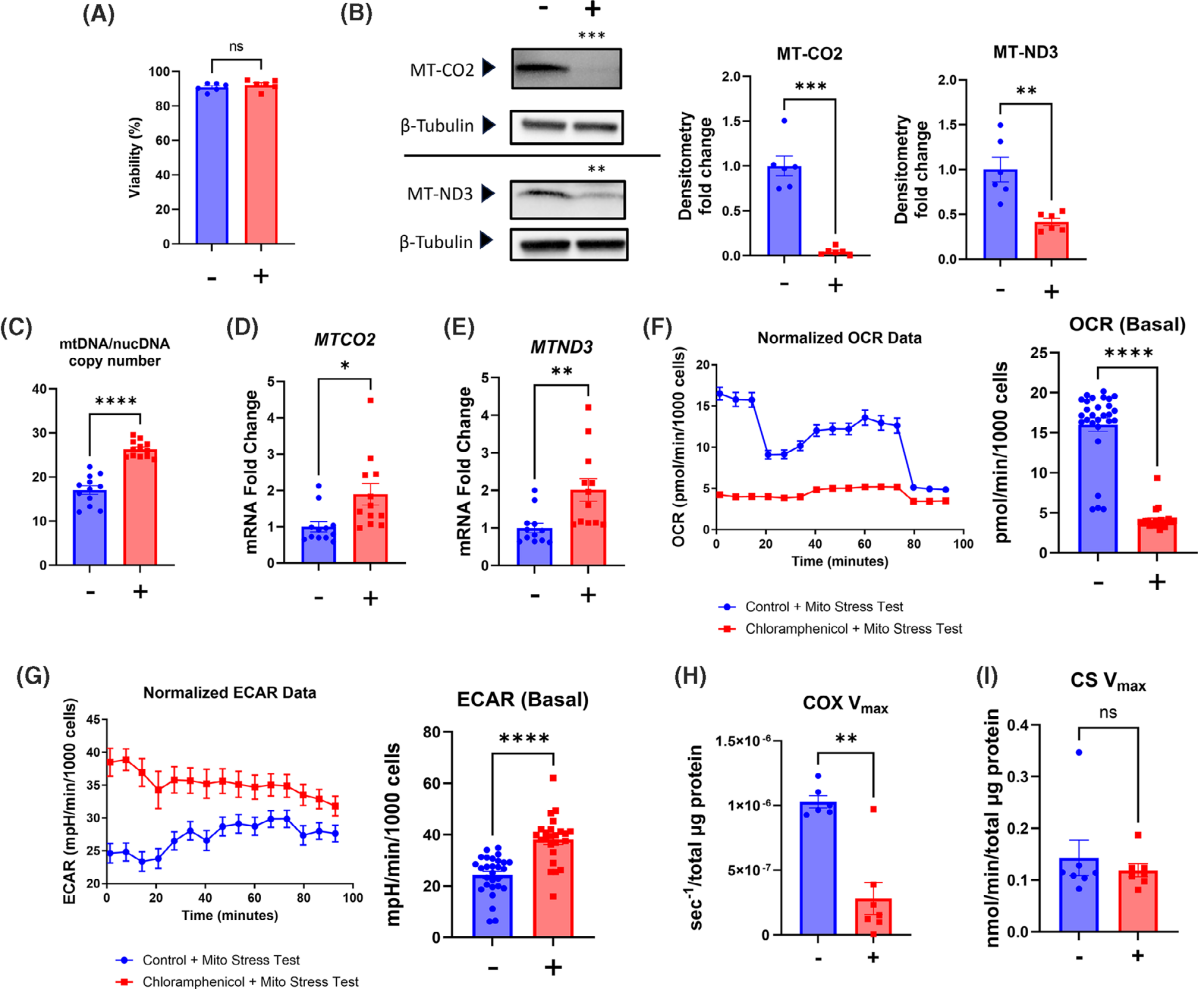
Chloramphenicol inhibits SH‐SY5Y ρ+ cell mtDNA transcript translation, induces compensatory mtDNA copy number and transcription changes, and alters bioenergetic flux and enzyme function. (A) Chloramphenicol untreated and treated cells appeared equally viable. (B) Treated cells contained less MT‐CO2 and MT‐ND3 protein. (C) Treated cells had a higher mtDNAcn. (D) Treated cells contained more *MTCO2* mRNA. (D) Treated cells contained more *MTND3* mRNA. (F) Chloramphenicol treatment reduced the basal OCR and blunted the response to ATP synthase inhibition and mitochondrial membrane permeabilization. (G) Chloramphenicol treatment increased the basal ECAR. (H) Treated cells had a lower COX V_max_ activity. (I) CS V_max_ activities were comparable. Statistical testing reflects two‐tailed Student's *t*‐tests except for the V_max_ data (H, I), which used Mann‐Whitney *U* tests. Error bars represent SEM. −/+ indicates with or without chloramphenicol treatment. COX, cytochrome c oxidase; CS, citrate synthase; ECAR, extracellular acidification rate; mtDNA, mitochondrial DNA; nucDNA, nuclear DNA; OCR, oxygen consumption rate; p+, with mtDNA; V_max_, maximal velocity. ns = not significant, *p ≤ 0.05, **p ≤ 0.01, ***p ≤ 0.001, ****p ≤ 0.0001.

To inhibit the translation of mtDNA‐expressed transcripts we exposed human SH‐SY5Y cells to chloramphenicol, an antibiotic that inhibits prokaryote ribosome function,^19^, ^21^ for 7 days. The dose and duration parameters we utilized did not reduce cell viability (Figure 1A). To verify target engagement, we assessed the status of two mtDNA‐encoded proteins, MT‐CO2 and MT‐ND3. Relative to untreated cells chloramphenicol treatment reduced the amount of both proteins (Figure 1B). We also screened for direct mitochondrial adaptations to and consequences of translation inhibition. The chloramphenicol treatment increased SH‐SY5Y cell mtDNA copy number (mtDNAcn), MT‐CO2 mRNA, and MT‐ND3 mRNA (Figure 1C‐E). Under basal conditions the chloramphenicol‐treated cells consumed less oxygen than the untreated control cells, showed blunted responses to ATP synthase inhibition with oligomycin and uncoupling with FCCP, and manifested a higher glycolysis flux as indicated by an elevated ECAR (Figure 1F, G). A COX V_max_ assay revealed an ≈85% reduction of the cytochrome c oxidation rate (Figure 1H), while the CS V_max_ rate did not change (Figure 1I).

**FIGURE 2:**
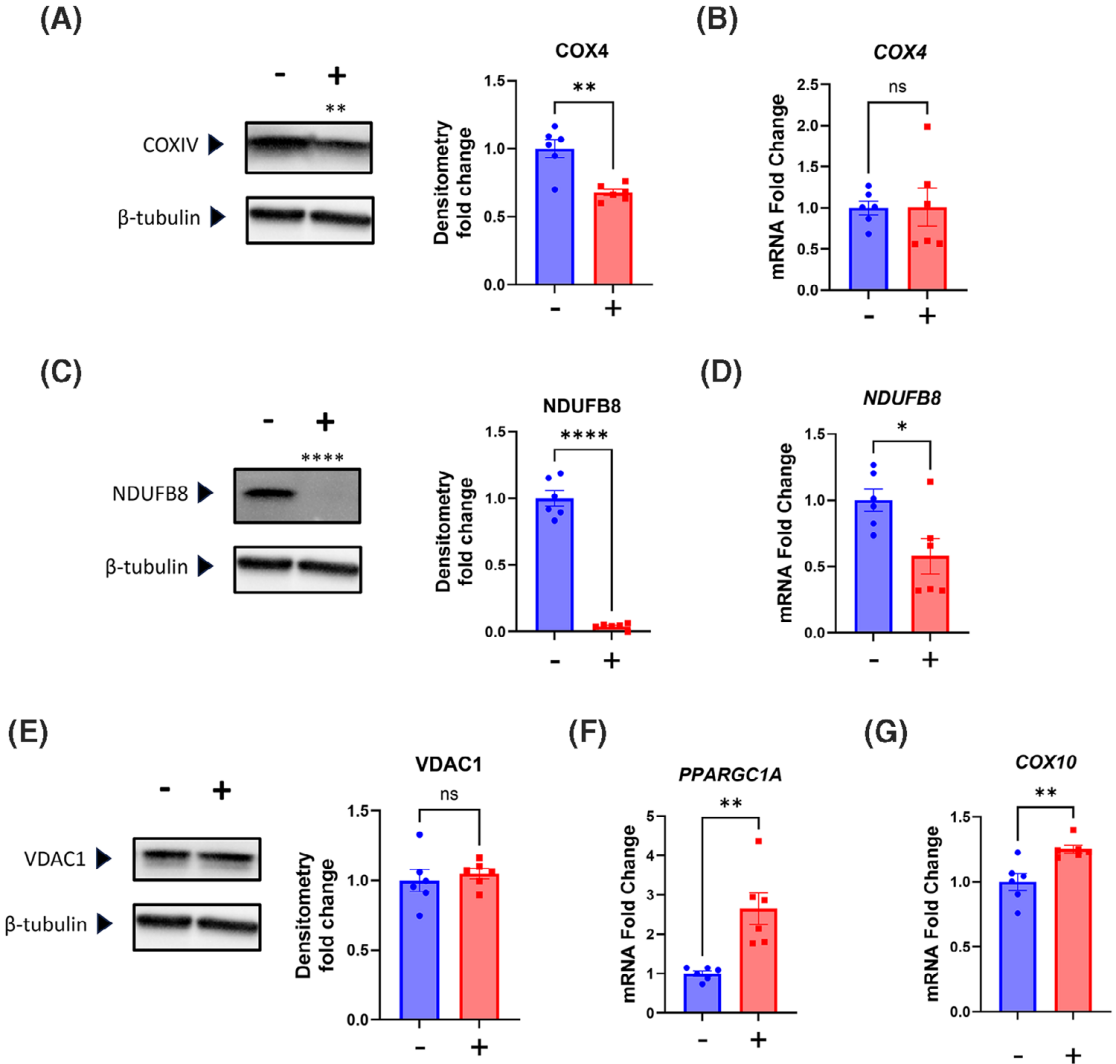
Effect of chloramphenicol on SH‐SY5Y ρ+ cell nuclear‐encoded, mitochondria‐associated genes, and proteins. (A) Chloramphenicol‐treated cells contained less COX4 protein. (B) *COX4* mRNA levels were comparable. (C) Treated cells contained less NDUFB8 protein. (D) *NDUFB8* mRNA was reduced in the treated cells. (E) VDAC1 protein levels were comparable. (F) *PPARGC1A* mRNA was higher in the treated cells. (G) *COX10* mRNA was unchanged. Statistical testing reflects two‐tailed Student's *t*‐tests. Error bars represent SEM. −/+ indicates with or without chloramphenicol treatment. COX, cytochrome c oxidase; p+, with mitochondrial DNA. ns = not significant, *p ≤ 0.05, **p ≤ 0.01, ***p ≤ 0.001, ****p ≤ 0.0001.

We considered chloramphenicol's influence on SH‐SY5Y cell nuclear‐encoded, mitochondria‐associated genes and proteins. Chloramphenicol reduced the amount of the COX4 respiratory chain subunit protein, while the COX4 mRNA level remained unchanged (Figure 2A,B). Protein and mRNA levels of the respiratory chain subunit NDUFB8 both declined (Figure 2C,D). The protein level of VDAC1, an outer mitochondrial membrane protein, did not change (Figure 2E). The amount of mRNA encoded by the *PPARGC1A* gene, which encodes a transcriptional co‐activator that facilitates mitochondrial biogenesis,^23^, ^24^ increased (Figure 2F). We also observed a significant increase in *COX10* mRNA, which encodes an enzyme involved in mitochondrial heme A biosynthesis and COX holoenzyme assembly.^25^

### 3.2 Chloramphenicol's impact on SH‐SY5Y cell AD‐Associated genes and proteins

**FIGURE 3:**
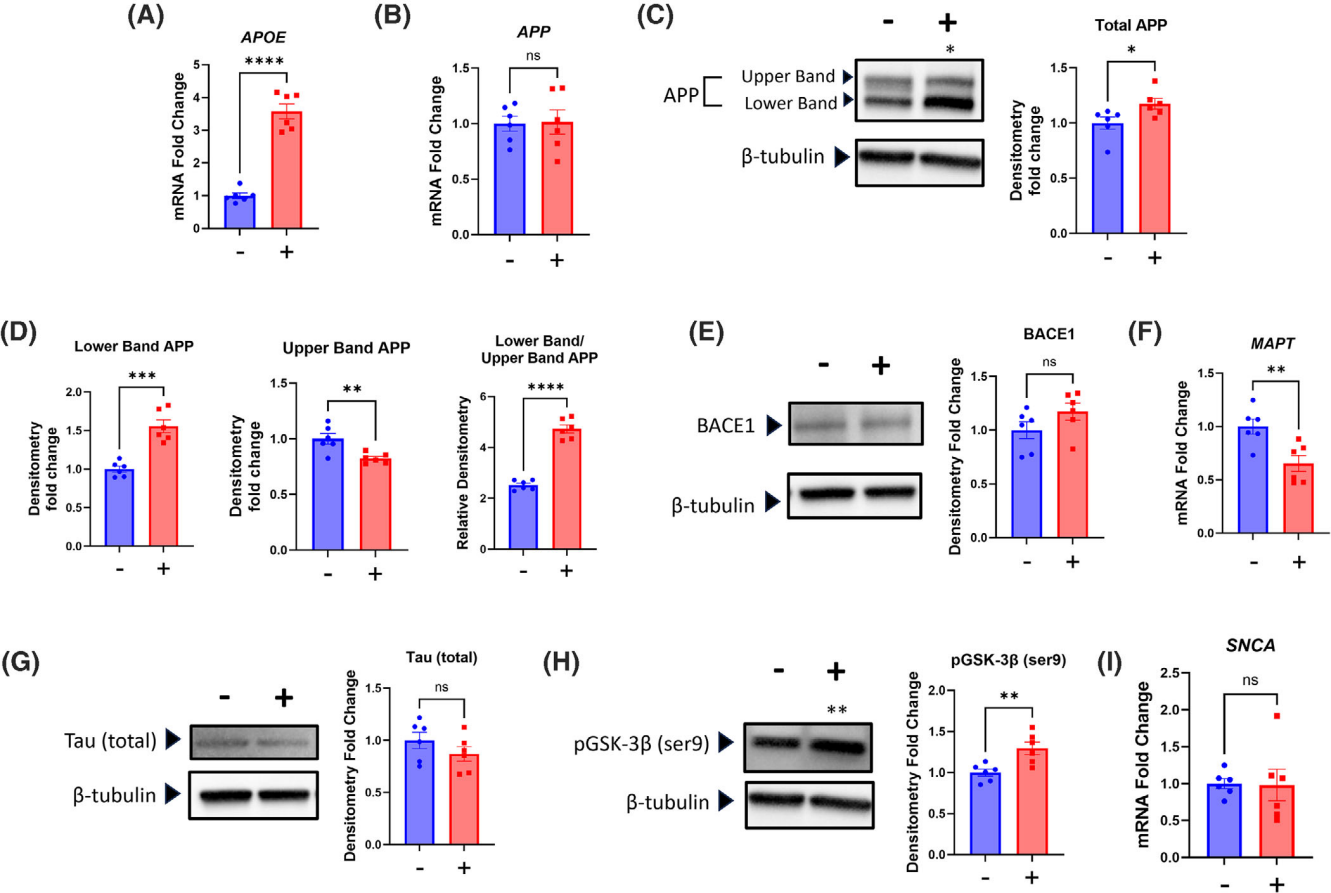
Chloramphenicol alters SH‐SY5Y ρ+ cell AD‐pertinent biology. (A) Chloramphenicol‐treated cells contained more *APOE* mRNA. (B) *APP* mRNA levels were comparable. (C) Treated cells contained more APP protein. (D) Treated cells contained more lower band APP, less upper band APP, and an increased lower band to upper band APP ratio. (E) BACE1 protein levels were comparable. (F) Treated cells contained less *MAPT* mRNA. (G) Western blot total tau protein levels were comparable. (H) Treated cells contained more ser9‐phosphorylated GSK‐3β. (I) *SNCA* mRNA levels were comparable. Statistical testing reflects two‐tailed Student's *t*‐tests. Error bars represent SEM. −/+ indicates with or without chloramphenicol treatment. AD, Alzheimer's disease; *APOE*, apolipoprotein E; APP, amyloid precursor protein; p+, with mitochondrial DNA; OCR, oxygen consumption rate;. ns = not significant, *p ≤ 0.05, **p ≤ 0.01, ****p ≤ 0.0001.

Chloramphenicol affected apoE/*APOE*, APP, and tau‐pertinent biology. The *APOE* mRNA level increased by 260% (Figure 3A). The *APP* mRNA level did not change, although the total amount of APP protein increased by 18% (Figure 3B,C). On western blots of SH‐SY5Y protein lysates full length APP presents as two bands, which could represent different splice isoforms or levels of glycosylation (Figure 3C).^26^ In this case the lower band drove the overall increase, as the density of the upper band was in fact slightly lower than it was in the control cells (Figure 3C,D). We used an ELISA kit to measure the amount of Aβ42 protein secreted into the medium but with the protocol we utilized, which did not specifically concentrate the protein content, the Aβ42 signal was below the limit of detection (data not shown). The level of BACE1 protein, which promotes the amyloidogenic processing of APP,^27^ did not change (Figure 3E). The *MAPT* mRNA level fell while the total tau protein level did not change, and the amount of GSK‐3β ser9‐phosphorylation, a post‐translational modification that increases its ability to phosphorylate tau,^28^ increased by 30% (Figure 3F‐H). We also assessed the level of tau serine 404 phosphorylation but did not detect a clear band (data not shown). The *SNCA* mRNA level did not change (Figure 3H). We attempted to measure the α‐synuclein protein level, but the western blot did not reveal a visible band (data not shown).

### 3.3 Validation of chloramphenicol's mechanism of action

**FIGURE 4:**
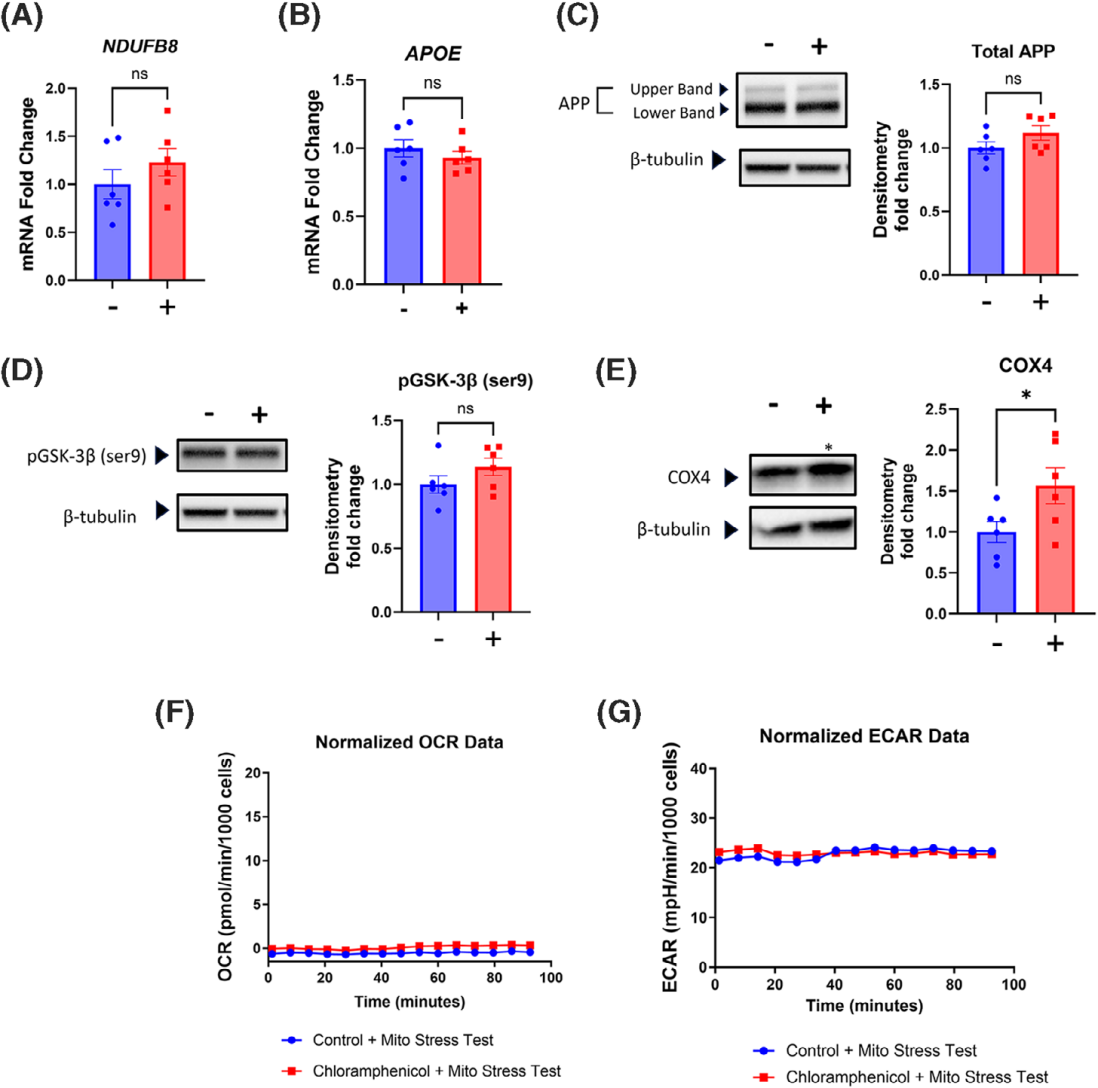
Chloramphenicol‐induced changes in SH‐SY5Y ρ+ cells are not observed in chloramphenicol‐treated SH‐SY5Y ρ0 cells. (A) *NDUFB8* mRNA levels were comparable between chloramphenicol treated and untreated SH‐SY5Y ρ0 cells. (B) *APOE* mRNA levels were comparable. (C) Total APP protein levels were comparable. (D) Ser9‐phosphorylated GSK‐3β levels were comparable. (E) Whereas chloramphenicol‐treated ρ+ cells contained less COX4 protein than their untreated controls, chloramphenicol‐treated SH‐SY5Y ρ0 cells contained more COX4 protein than their untreated controls. (F) OCRs were essentially undetectable and non‐responsive to both ATP synthase inhibition and mitochondrial membrane permeabilization. (G) ECARs were comparable. Statistical testing reflects two‐tailed Student's *t*‐tests. Error bars represent SEM. −/+ indicates with or without chloramphenicol treatment. *APOE*, apolipoprotein E; APP, amyloid precursor protein; COX, cytochrome c oxidase; ECAR, extracellular acidification rate; p+, with mitochondrial DNA; ρ0, without mitochondrial DNA. ns = not significant, *p ≤ 0.05.

To verify that inhibition of mtDNA transcript translation mediated chloramphenicol's effects on SH‐SY5Y ρ+ cells, we treated SH‐SY5Y ρ0 cells with chloramphenicol for an equivalent duration and at the same concentration. ρ0 cells lack mtDNA, do not express mtDNA‐derived transcripts, and cannot generate mitochondrial ribosomes.^20^ Parameters that changed in the parent SH‐SY5Y cell line did not change in the ρ0 cells, including *NDUFB8* mRNA, *APOE* mRNA, total APP protein, and GSK‐3β ser9‐phosphorylation (Figure 4A‐D). The COX4 subunit protein level increased, but this change was opposite in direction to the COX4 decrease observed in the ρ+ cells (Figure 4E; compare to Figure 2A). The ρ0 cell mitochondria consumed essentially no oxygen with or without chloramphenicol, and the ECARs were comparable (Figure 4F, G).

### 3.4 Chloramphenicol's impact on iPSC‐derived neuron mitochondria and adaptive responses

**FIGURE 5:**
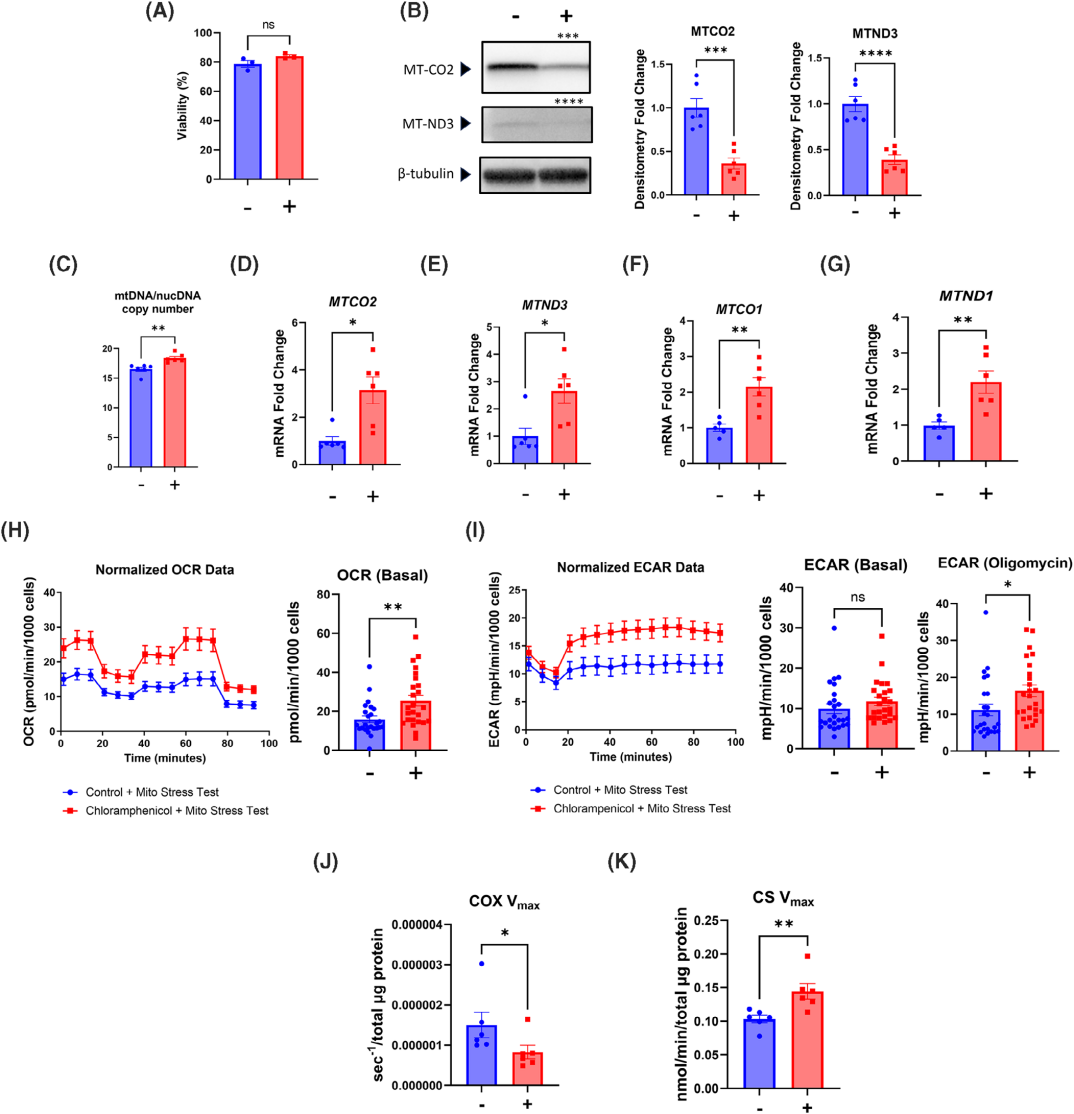
Chloramphenicol inhibits iPSC‐derived neuron mtDNA transcript translation, induces compensatory mtDNA copy number and transcription changes, and alters bioenergetic flux and enzyme function. (A) Chloramphenicol untreated and treated neurons appeared equally viable. (B) Treated neurons contained less MT‐CO2 and MT‐ND3 protein. (C) Treated neurons had a higher mtDNAcn. (D) Treated neurons contained more *MTCO2* mRNA. (E) Treated neurons contained more *MTND3* mRNA. (F) Treated neurons contained more *MTCO1* mRNA. (G) Treated neurons contained more *MTND1* mRNA. (H) Chloramphenicol treatment increased the basal OCR. (I) Although the basal ECARs were comparable, following ATP synthase inhibition the ECAR was higher in the chloramphenicol‐treated neurons. (J) Treated neurons had a lower COX V_max_ activity. (K) The CS V_max_ activity was higher in the treated neurons. Statistical testing reflects two‐tailed Student's *t*‐tests except for the V_max_ data (J, K), which used Mann‐Whitney *U* tests. Error bars represent SEM. −/+ indicates with or without chloramphenicol treatment. COX, cytochrome c oxidase; CS, citrate synthase; ECAR, extracellular acidification rate; iPSC, induced pluripotent stem cell; mtDNA, mitochondrial DNA; OCR, oxygen consumption rate; V_max_, maximal velocity. ns = not significant, *p ≤ 0.05, **p ≤ 0.01, ***p ≤ 0.001, ****p ≤ 0.0001.

We extended our studies to human iPSC‐derived forebrain neurons. The dose and duration parameters we used did not reduce cell viability (Figure 5A). Reflective of the SH‐SY5Y ρ+ cells, chloramphenicol treatment reduced MT‐CO2 and MT‐ND3 protein levels, increased the mtDNAcn, and increased the amount of MTCO2 and MTND3 mRNA (Figures 5B‐E). For the iPSC‐derived neurons we additionally showed MTCO1 and MTND1 mRNA levels increased (Figure 5F,G). Contrary to our findings in SH‐SY5Y ρ+ cells, the basal OCR increased with chloramphenicol treatment, and we observed responses to oligomycin and FCCP (Figure 5H). Chloramphenicol did not increase the basal ECAR, which also deviates from our SH‐SY5Y ρ+ cell data, and after the addition of oligomycin the ECAR rose more in the treated neurons than it did in the untreated neurons (Figure 5I). The COX V_max_ activity was significantly reduced in the treated neurons (Figure 5J), although from a proportional perspective this 40% decline appeared less robust than the 85% decline observed with the SH‐SY5Y ρ+ cells (compare to Figure 1H). Unlike the SH‐SY5Y ρ+ cells, chloramphenicol treatment increased the neuron CS V_max_ activity (Figure 5K).

**FIGURE 6:**
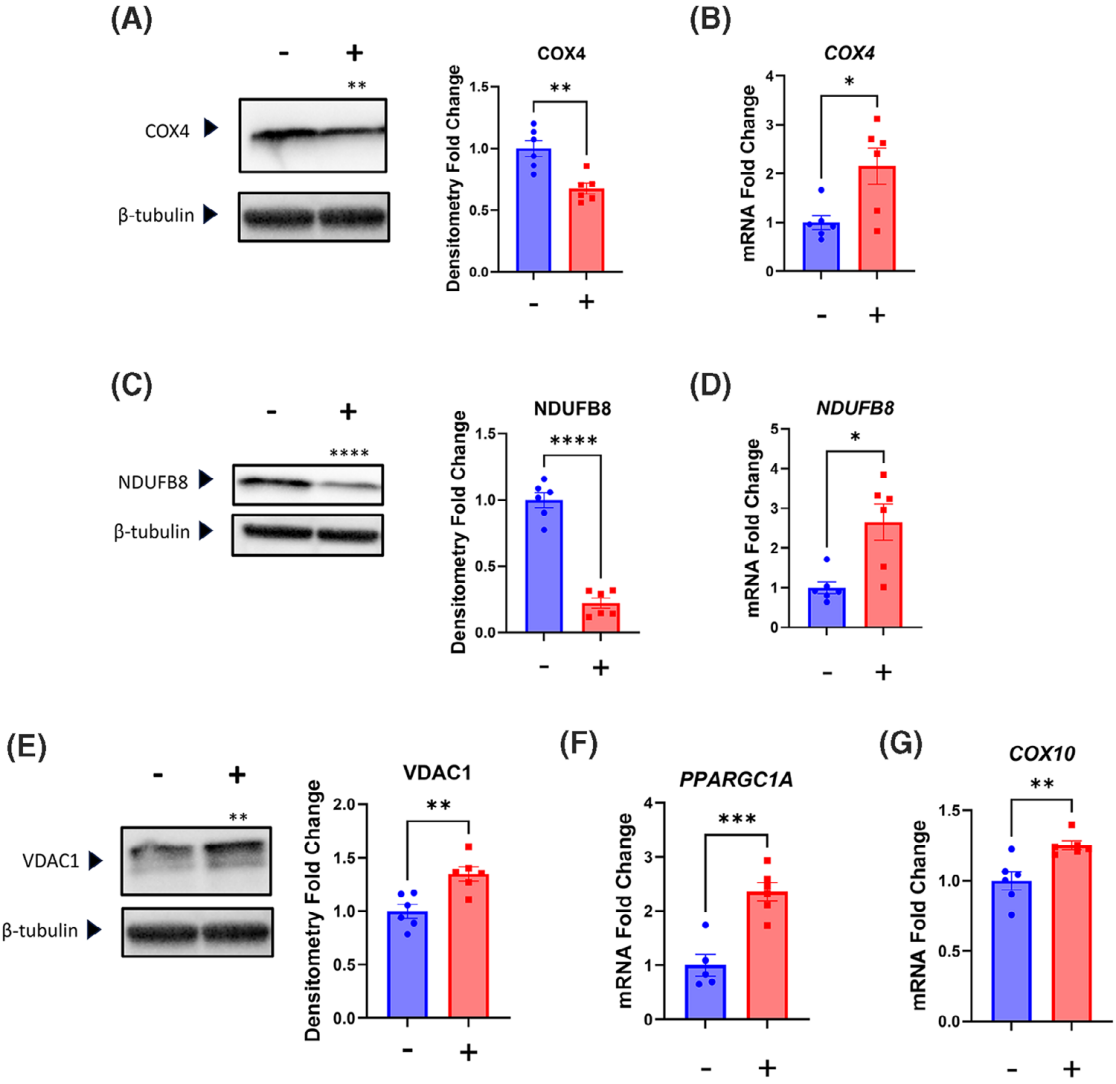
Effect of chloramphenicol on iPSC‐derived neuron nuclear‐encoded, mitochondria‐associated genes and proteins. (A) Chloramphenicol‐treated neurons contained less COX4 protein. (B) Treated neurons contained more *COX4* mRNA. (C) Treated neurons contained less NDUFB8 protein. (D) Treated neurons contained more *NDUFB8* mRNA. (E) Treated neurons contained more VDAC1 protein. (F) *PPARGC1A* mRNA was higher in the treated neurons. (G) *COX10* mRNA was higher in the treated neurons. Statistical testing reflects two‐tailed Student's *t*‐tests. Error bars represent SEM. −/+ indicates with or without chloramphenicol treatment. COX, cytochrome c oxidase; iPSC, induced pluripotent stem cell. ns = not significant, *p ≤ 0.05, **p ≤ 0.01, ***p ≤ 0.001, ****p ≤ 0.0001.

Reflective of the SH‐SY5Y ρ+ cells, neuron COX4 protein decreased, but unlike what we observed with the SH‐SY5Y ρ+ cells neuron COX4 mRNA increased (Figure 6A, B). NDUFB8 protein fell as it did in the SH‐SY5Y ρ+ cells, but opposite to those cells the neurons increased their NDUFB8 mRNA (Figure 6C, D). Whereas the SH‐SY5Y ρ+ cell VDAC1 protein level did not change, chloramphenicol increased neuron VDAC1 protein (Figure 6E). The chloramphenicol‐treated neurons increased their *PPARGC1A* mRNA (Figure 6F). The amount of mRNA encoded by the *COX10* gene also increased (Figure 6G).

### 3.5 Chloramphenicol's impact on iPSC‐derived neuron AD‐associated genes and proteins

**FIGURE 7:**
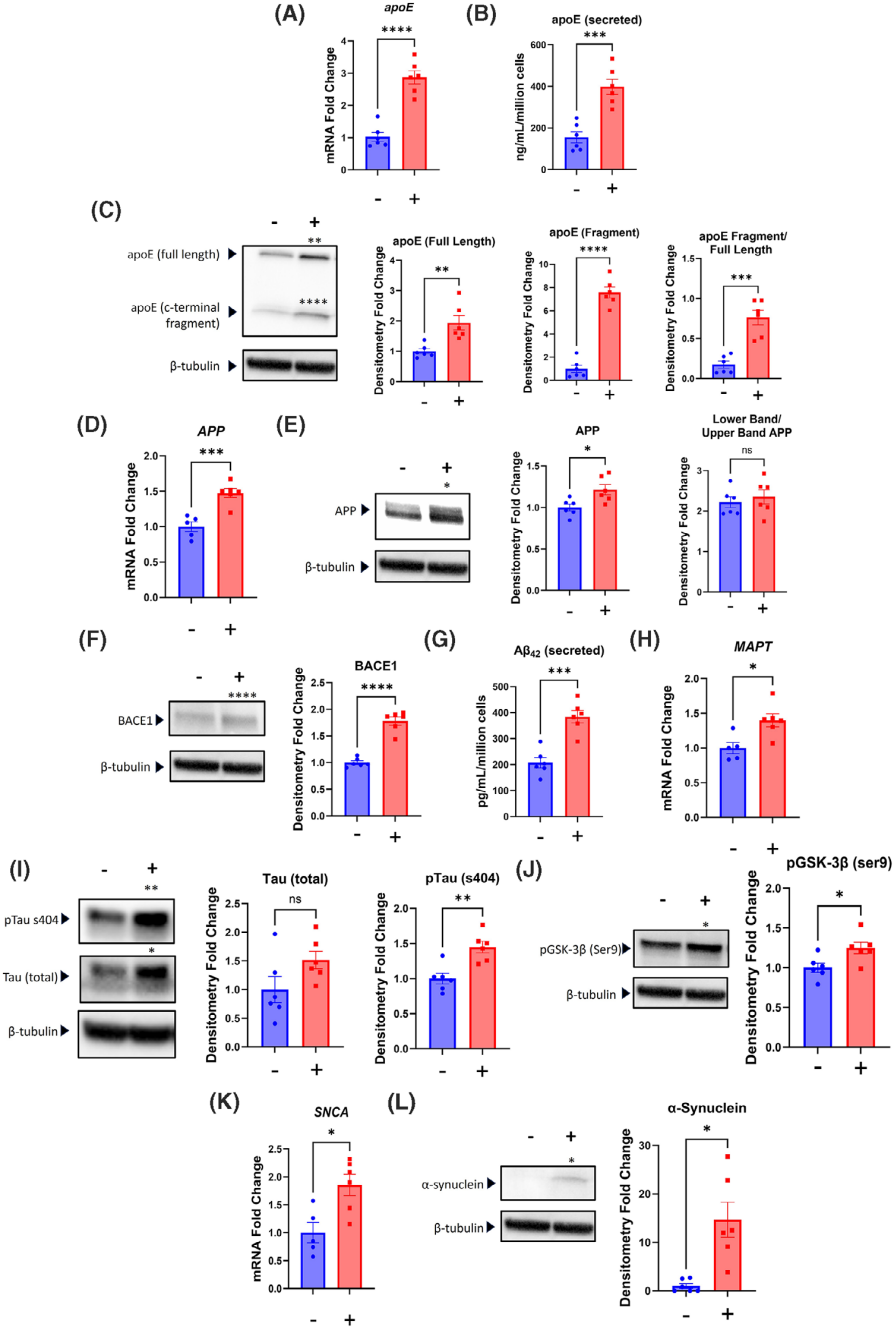
Chloramphenicol alters iPSC‐derived neuron AD‐pertinent biology. (A) Chloramphenicol‐treated neurons contained more *APOE* mRNA. (B) An apoE ELISA showed the amount of apoE protein was higher in the media of the treated neurons. (C) The amount of apoE protein was higher in the treated neuron lysates. On western blot this manifested as an increase in the full‐length protein, and as an increase in an ∼12 kD band that reportedly represents a C‐terminal apoE fragment. The fragment to full length ratio was higher in the treated neurons. (D) The *APP* mRNA level was higher in the treated neurons. (E) Treated neurons contained more APP protein, but unlike what was observed with the SH‐SY5Y ρ+ cells the neuronlower band to upper band APP ratios were comparable. (F) BACE1 protein levels were higher in the treated neurons. (G) An ELISA for Aβ42 showed the amount of Aβ42 protein was higher in the media of the treated neurons. (H) Treated cells contained more *MAPT* mRNA. (I) Western blot total tau and serine 404‐phosphorylated tau protein levels were higher in the treated neurons. (J) Treated neurons contained more serine 9‐phosphorylated GSK‐3β. (K) *SNCA* mRNA levels were higher in the treated neurons. (L) The treated neurons contained more α‐synuclein protein. Statistical testing reflects two‐tailed Student's *t*‐tests. Error bars represent SEM. −/+ indicates with or without chloramphenicol treatment. Aβ, beta amyloid; AD, Alzheimer's disease; apoE/*APOE*, apolipoprotein E; APP, amyloid precursor protein; ELISA, enzyme‐linked immunoassay; iPSC, induced pluripotent stem cell; p+, with mitochondrial DNA. ns = not significant, *p ≤ 0.05, **p ≤ 0.01, ****p ≤ 0.0001.

As was the case with the SH‐SY5Y ρ+ cells, chloramphenicol affected neuron apoE/*APOE*, APP, and tau biology. *APOE* mRNA increased (Figure 7A). The apoE protein is typically secreted, and the amount of apoE present in the medium increased by 160% (Figure 7B). The amount of full‐length apoE in the neurons themselves doubled, levels of a smaller ∼12 kD band that could represent a C‐terminal derivative increased by 700%,^29^ and the fragment to full length apoE protein ratio increased (Figure 7C). *APP* mRNA and total APP protein both increased. Although two APP bands were apparent, the separation of these bands was not as distinct as it was in the SH‐SY5Y cells, and their ratio did not change (Figure 7D,E). The BACE1 protein level increased by 80% (Figure 7F). The amount of Aβ42 present in the media surpassed the detection threshold of our ELISA kit, and 85% more Aβ42 was found in the media from the chloramphenicol‐treated neurons (Figure 7G). *MAPT* mRNA, total tau protein, serine 404 phosphorylated tau protein, and serine 9 phosphorylated GSK‐3β protein each increased (Figure 7H‐J). The *SNCA* mRNA level nearly doubled, and there was a 14.5‐fold increase in the amount of α‐synuclein protein (Figure 7K,L).

**FIGURE 8:**
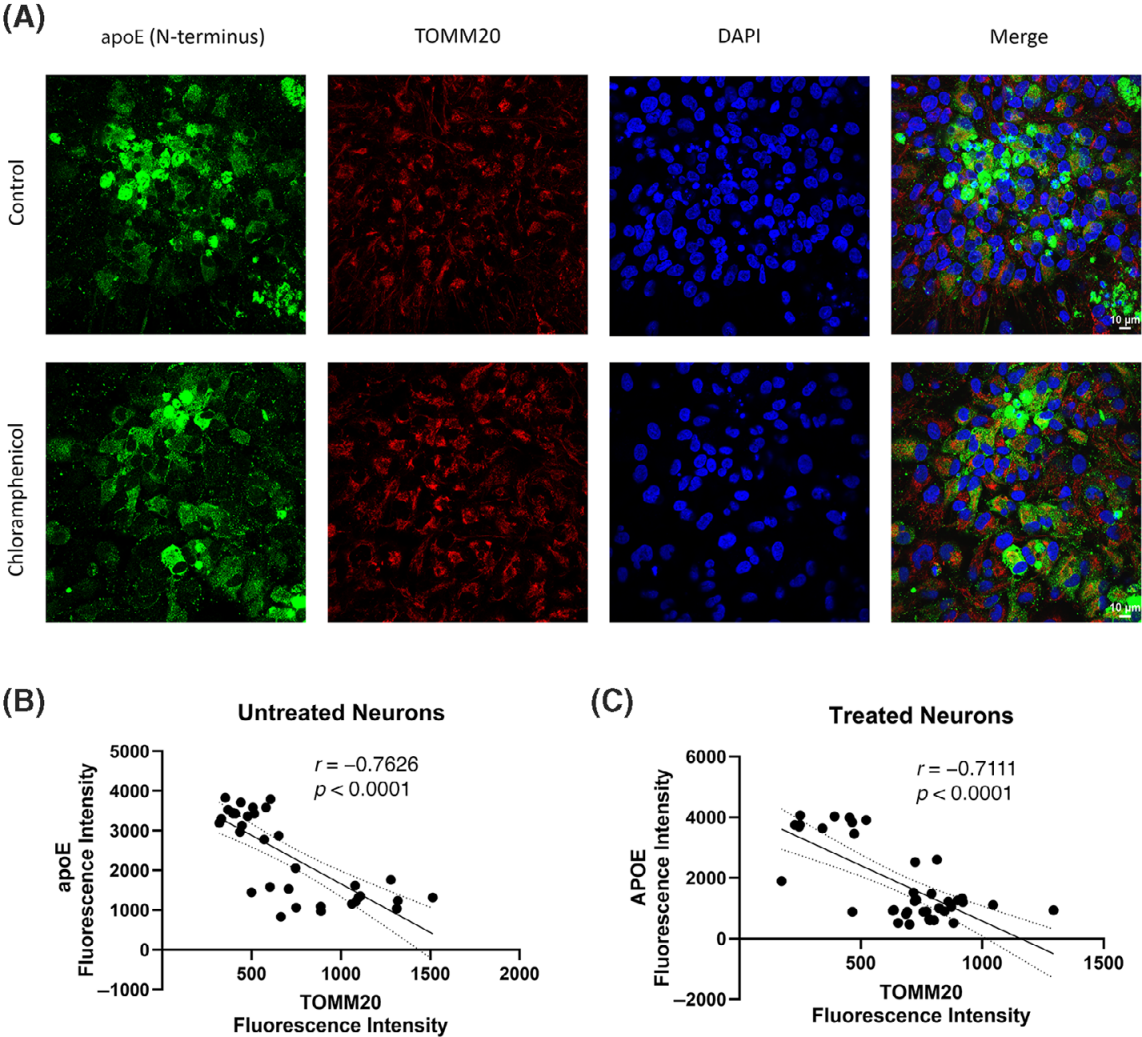
Inverse correlation between apoE and TOMM20 protein levels in iPSC‐derived neurons. (A) Representative confocal images of chloramphenicol‐treated and untreated neurons stained with an N‐terminus region apoE antibody (green), TOMM20 antibody (red), and DAPI. (B) In the untreated condition, individual neurons showed an inverse relationship between apoE and TOMM20 protein levels; the Pearson correlation coefficient was −0.7626. (C) In the treated condition, individual neurons showed an inverse relationship between apoE and TOMM20 protein levels; the Pearson correlation coefficient was −0.7111. In (B) and (C), the solid line represents the linear regression, while the dashed lines represent the 95% confidence intervals. apoE, apolipoprotein E; DAPI, 4′,6‐diamidino‐2‐phenylindole; iPSC, induced pluripotent stem cell.

We placed control and chloramphenicol‐treated neurons on coverslips, stained with antibodies directed to TOMM20 and the apoE N‐terminal region, and analyzed the TOMM20 and apoE immunofluorescence levels within individual neurons (Figure 8A). We observed a robust inverse correlation between the amount of neuron apoE and TOMM20 proteins. This relationship was present in both the control and chloramphenicol‐treated conditions. Neurons with very intense apoE staining generally showed very low amounts of TOMM20 staining (Figure 8B,C).

## 4 DISCUSSION

We inhibited translation of mtDNA‐encoded transcripts to induce a state of sustained primary mitochondrial dysfunction in human neuronal cells and iPSC‐derived neurons. To do this we used chloramphenicol, an antibiotic that inhibits mitochondrial ribosome function.^19^, ^21^, ^30^, ^31^ Reduced mtDNA‐encoded protein levels verified target engagement, and the failure of SH‐SY5Y ρ0 cells to manifest changes observed in SH‐SY5Y ρ+ cells demonstrates target specificity.

Neuronal cells and neurons remained viable for at least 1 week, a period long enough to initiate molecular adaptations. After 1 week the ρ+ models exhibited intra‐ and extra‐mitochondrial adaptive changes. During this time, we observed neither excess nor excessive cell death, which suggests these responses reflect pro‐survival and not agonal events.

Some assessments suggest chloramphenicol‐treated neuronal cells and neurons adjusted to increase mitochondrial mass. Shared examples include increased mtDNAcn, mtDNA transcription, and *PPARGC1A* expression. The neurons also increased their VDAC1 protein, OCR, nuclear‐encoded respiratory chain mRNA expression, and CS V_max_ activity. Despite this, both models contained reduced nuclear‐encoded respiratory chain proteins. In the chloramphenicol‐treated neurons, discordance between levels of nuclear‐encoded respiratory chain gene mRNAs (increased) and their corresponding proteins (decreased) suggests inefficient translation of those mRNAs or an inability to preserve their protein products.

Several factors could explain the divergence in OCR responses. Replicating, tumor‐derived cells and non‐replicating, non‐tumor‐derived cells experience different bioenergetic and biosynthetic demands and meet those demands through different strategies. Tumor cells depend less on respiration to produce ATP and divert acetyl CoA carbon to macromolecule synthesis.^32^ Stress‐induced changes to the tumor cell replication cycle present an additional variable. Toxin sensitivity and resilience may also differ between SH‐SY5Y and iPSC‐derived cells. To this point, chloramphenicol‐treated neurons consistently increased nuclear‐encoded respiratory chain mRNAs while in chloramphenicol‐treated SH‐SY5Y ρ+ cells levels either decreased or did not change. Perhaps our protocol produced less inhibition of neuron mtDNA transcript translation and left enough residual neuron COX activity to meet the increased bioenergetic needs of a stressed system. Alternatively, SH‐SY5Y ρ+ cells perhaps simply relied on upregulated glycolysis to meet energy demand. Our data support either or both scenarios.

We used a fibroblast‐derived iPSC line, and the OCR and ECAR shifts in our chloramphenicol‐treated neurons reflect those reported in a study of AD subject‐derived fibroblasts.^33^ That study's authors noted fibroblasts, like neurons, have a limited ability to boost glycolysis flux in the setting of inefficient respiration,^34^ which could elevate the OCR. It is worth considering this phenomenon's potential impact on our chloramphenicol‐treated neuron model, which experienced stress across various systems.

Changes in nuclear gene mRNAs emphasize the relevance of mitochondria‐nuclear crosstalk. Various mechanisms, including but not limited to oxidative stress, acetyl CoA trafficking, a mitochondrial unfolded protein response (mtUPR), and the integrated stress response (ISR) reportedly mediate this communication.^35^, ^36^, ^37^, ^38^ The mechanism used by a particular system appears situation, cell type, or species dependent.^39^ Elucidating the mediators of mitochondria‐nuclear crosstalk could inform our understanding of brain health, as the mitochondria's ability to initiate or amplify changes in nuclear gene expression are arguably relevant to brain aging and age‐related diseases such as AD.^39^

Mitochondria impact AD phenomena,^2^, ^7^ and genetic variation in the *PTCD1* gene, which encodes a mitochondrial translation‐relevant peptide, associates with AD risk.^17^ Our current study used toxin‐induced mitochondrial translation inhibition to create a state of sustained, primary mitochondrial dysfunction and data from our models confirm mitochondrial function affects AD phenomena. Our models demonstrate inhibiting mitochondrial translation alters apoE/*APOE*, APP, tau, and α‐synuclein gene expression, protein levels, or protein handling.

Genetic studies link *APOE* to AD, but the responsible mechanisms remain unclear.^40^, ^41^, ^42^ One study found brain *APOE* mRNA increases during the mild cognitive impairment (MCI) syndrome that represents the first objectively detectable stage of AD cognitive decline,^43^ while another reported increased *APOE* mRNA in demented AD patient brains.^44^ We and others previously showed other types of primary mitochondrial dysfunction increase neuronal and glial cell *APOE* expression.^16^, ^45^ Our current study adds mitochondrial translation inhibition to this list. In the brain astrocytes typically express *APOE* and secrete apoE protein. We found that although neurons also secrete the apoE they produce, intraneuronal apoE and apoE‐derived peptides also accumulate, especially in neurons with reduced TOMM20 protein. The purpose of neuron‐expressed apoE protein in its secreted and retained forms remains unclear, although it is known drosophila neurons express an apoE ortholog that shuttles oxidized neuron lipids to astrocytes.^46^

Genetic data link *APP* to AD, and APP cleavage produces Aβ peptides that aggregate to form amyloid plaques.^47^ Persons with Down syndrome contain an additional *APP* copy and experience accelerated plaque accumulation.^48^ Chloramphenicol‐treated SH‐SY5Y ρ+ cells and iPSC neurons contained more APP protein than untreated cells, and *APP* mRNA also increased in treated neurons. Following its synthesis APP traffics through the Golgi, where it glycosylates.^49^, ^50^ If differences in APP glycosylation contribute to the APP band separation in SH‐SY5Y cells, the shift in their APP ratio could reflect a fundamental problem with glycosylation or altered APP trafficking. Additional studies could address these possibilities. In the neurons we also observed an increase in BACE1, the enzyme that cleaves APP to initiate its processing to Aβ, and the amount of secreted Aβ. The observed increases in APP, BACE1, and secreted Aβ protein suggest chloramphenicol increased neuron Aβ production.

Tau protein forms the neurofibrillary tangles that accumulate in neurons in AD.^51^ Levels of the *MAPT*‐encoded tau mRNA, total tau protein, and a phosphorylated tau species (serine 404) increased in our chloramphenicol‐treated neurons. Tau phosphorylation associates with tangle deposition, and the GSK‐3β enzyme phosphorylates tau, especially when it is phosphorylated at serine 9.^28^ Phosphorylated tau serine 404 levels increase in early AD and Down Syndrome brains.^52^ Chloramphenicol‐treated neurons contained more phosphorylated GSK‐3β protein. Such changes could facilitate tangle formation.

α‐synuclein aggregates to form insoluble Lewy bodies that pathologically define Parkinson's disease and Lewy body dementias.^53^, ^54^ α‐synuclein and Aβ deposition frequently co‐exist in cognitively impaired individuals.^55^, ^56^ Chloramphenicol increased neuron α‐synuclein mRNA and protein. Increasing a protein's intracellular concentration may drive its transition from a soluble, unaggregated to insoluble, aggregated state.^57^, ^58^

Many of the apoE/*APOE*, APP, tau, and α‐synuclein changes we report suggest impaired mitochondrial translation promotes the development of AD‐associated pathology. Some of our data, though, support less straightforward or potentially contradictory interpretations. For example, AD patient autopsy brains show lower mtDNAcn and respiratory chain gene expression than control patient brains,^59^, ^60^, ^61^ while both our models showed increased mtDNAcn relative to the control condition and the neurons showed elevated respiratory chain gene mRNA levels. To this point, perhaps we induced an insufficient duration or degree of mitochondrial dysfunction. Human and animal data reveal mixed and often contradictory trends in the direction of mtDNAcn changes in both advancing age and disease.^62^, ^63^, ^64^ In healthy older individuals, brain mtDNAcn may increase, which likely represents a compensatory response to an age‐associated decline in mtDNA transcription efficiency.^65^ Given this context, it is difficult to know whether lower mtDNAcn in AD brains reflects a decline from a previously obtained baseline, or an inability to drive mtDNAcn to a level required to maintain mitochondrial function. As the study of Hirai et al. points out, in AD brains mitochondrial mass and content change in complex ways, as surviving neurons may contain increased amounts of total, likely mutated mtDNA and respiratory chain protein.^66^

AD patient cerebrospinal fluid (CSF) contains less Aβ than control subject CSF.^67^ This could reflect diversion of secreted Aβ into plaques, but other explanations warrant consideration. Wilkins et al. recently reported SH‐SY5Y ρ0 cells, which do not respire, redirect APP away from the plasma membrane and secrete less Aβ than SH‐SY5Y ρ+ cells.^68^ An increase in mitochondrial oxygen consumption and increase in Aβ secretion by our chloramphenicol‐treated neurons stress the need to evaluate higher chloramphenicol concentrations and longer exposures.

Many antibiotics affect mitochondria. For example, tetracycline impacts mitochondrial translation,^69^ and in the in vitro setting tetracycline may directly mitigate Aβ fibrillization.^70^ Elderly individuals taking tetracyclines may have a reduced dementia incidence.^71^ Findings such as these emphasize the general challenge of human disease modelling.

Descriptive human studies define what, when, and where changes occur in AD but do not address how and why characteristic pathologies arise. Resolving these how and why questions requires molecular manipulations we can only achieve through modeling. Many investigators rely on models that feature expression of mutated APP or the direct exposure of a system to Aβ, but these models cannot test predictions made by a mitochondrial cascade hypothesis that proposes primary mitochondrial dysfunction initiates AD.^7^, ^8^ We previously reported AD‐relevant studies in which we achieved states of primary mitochondrial dysfunction via the transfer of AD subject mtDNA to ρ0 cells to create cytoplasmic hybrid (cybrid) cell lines, and more recently by using ρ0 cells themselves.^1^, ^12^, ^13^, ^14^, ^16^, ^72^ The mitochondrial translation inhibition model we now describe will facilitate studies that advance our understanding of mitochondria‐nuclear communication and inform the integrity of the mitochondrial cascade hypothesis.

## CONFLICT OF INTEREST STATEMENT

The authors have no conflict of interest or competing interests to report. Author disclosures are available in the supporting information.

## CONSENT STATEMENT

There are no human subjects in this study.

## Supporting information



Supporting Information

Supporting Information
