# Supplementary material for: Inhibiting mtDNA transcript translation alters Alzheimer's disease‐associated biology
Source: Alzheimers Dement. 2024 Oct 23;20(12):8429–43. doi: 10.1002/alz.14275 (PMC11667520; doi:10.1002/alz.14275)
Supplement: Supplementary file 1 — Supporting Information [file ALZ-20-8429-s002.docx]

**SUPPLEMENTARY INFORMATION**

| **Table S1. Antibodies used for western blot immunochemistry.** | | | |
| --- | --- | --- | --- |
| **Antibody Name/Target** | **Dilution** | **Source** | **Catalog #** |
| APOE (C-terminus) | 1:1000 | Abcam | 52607 |
| APP | 1:1000 | Cell Signaling Technology | 76600 |
| BACE1 | 1:1000 | Abcam | 108394 |
| COX4 | 1:1000 | Cell Signaling Technology | 4844 |
| Goat anti-Mouse IgG (H+L), Superclonal Recombinant Secondary Antibody, HRP | 1:3500 | Invitrogen | A28177 |
| Goat anti-Rabbit IgG (Heavy Chain), Superclonal Recombinant Secondary Antibody, HRP | 1:3500 | Invitrogen | A27036 |
| MTCO2 | 1:1000 | abcam | 91317 |
| MTND1 | 1:1000 | Proteintech | 19703-1-AP |
| MTND3 | 1:1000 | Abcam | 170681 |
| NDUFB8 | 1:2000 | Abcam | 110242 |
| NDUFB8 | 1:1000 | CST | 73951 |
| Phospho-GSK-3β (Ser9) | 1:2000 | Cell Signaling Technology | 5558 |
| Phospho-Tau (s404) | 1:2000 | Abcam | 92676 |
| Tau (total) | 1:2000 | Abcam | 76128 |
| VDAC1 | 1:1000 | Abcam | 15895 |
| α-synuclein | 1:10000 | Abcam | 138501 |
| β-Tubulin | 1:2000 | Cell Signaling Technology | 2146 |

| **Table S2. Antibodies used for ICC.** | | | |
| --- | --- | --- | --- |
| **Antibody Name/Target** | **Dilution** | **Source** | **Catalog #** |
| APOE (N-terminus) | 1:100 | Abcam | 51015 |
| Donkey anti mouse Alexa 546 conjugated | 1:300 | Invitrogen | A10036 |
| Donkey anti rabbit Alexa 488 conjugated | 1:300 | Jackson Immunoresearch labs | 711-545-152 |
| TOMM20 | 1:300 | Abcam | 56783 |

| **Table S3. Taqman primers used for quantitative PCR.** | |
| --- | --- |
| **Primer Gene Target** | **Assay ID #** |
| *ACTB* | Hs99999903_m1 |
| *APOE* | Hs00171168_m1 |
| *APP* | Hs00169098_m1 |
| *COX10* | Hs01093077_m1 |
| *MAPT* | Hs00902194_m1 |
| *MTCO1* | Hs02596864_g1 |
| *MTND1* | Hs02596873_s1 |
| *PPARGC1A* | Hs01016719_m1 |
| *SNCA* | Hs00240906_m1 |
| *MTCO2* | Hs02596865_g1 |
| *MTND3* | Hs02596875_s1 |
| *COX4\|1* | Hs00971639_m1 |
| *NDUFB8* | Hs00428204_m1 |
